# Neighborhood Food Outlets, Diet, and Obesity Among California Adults, 2007 and 2009

**DOI:** 10.5888/pcd10.120123

**Published:** 2013-03-14

**Authors:** Aiko Hattori, Ruopeng An, Roland Sturm

**Affiliations:** Author Affiliations: Ruopeng An, Pardee RAND Graduate School, Santa Monica, California; Roland Sturm, RAND Corporation, Santa Monica, California.

## Abstract

**Introduction:**

Varying neighborhood definitions may affect research on the association between food environments and diet and weight status. The objective of this study was to examine the association between number and type of neighborhood food outlets and dietary intake and body mass index (BMI) measures among California adults according to the geographic size of a neighborhood or food environment.

**Methods:**

We analyzed data from 97,678 respondents aged 18 years or older from the 2007 and 2009 California Health Interview Survey through multivariable regression models. Outcome variables were BMI, weight status of a BMI of 25.0 or more and a BMI of 30.0 or more, and the number of times per week the following were consumed: fruits, vegetables, sugar-sweetened soft drinks, fried potatoes, and fast food. Explanatory variables were the number of fast-food restaurants, full-service restaurants, convenience stores, small food stores, grocery stores, and large supermarkets within varying distances (0.25 to 3.0 miles) from the survey respondent’s residence. We adopted as a measure of walking distance a Euclidean distance within 1 mile. Control variables included sociodemographic and economic characteristics of respondents and neighborhoods.

**Results:**

Food outlets within walking distance (≤1.0 mile) were not strongly associated with dietary intake, BMI, or probabilities of a BMI of 25.0 or more or a BMI of 30.0 or more. We found significant associations between fast-food outlets and dietary intake and between supermarkets and BMI and probabilities of a BMI of 25.0 or more and a BMI of 30.0 or more for food environments beyond walking distance (>1.0 mile).

**Conclusion:**

We found no strong evidence that food outlets near homes are associated with dietary intake or BMI. We replicated some associations reported previously but only for areas that are larger than what typically is considered a neighborhood. A likely reason for the null finding is that shopping patterns are weakly related, if at all, to neighborhoods in the United States because of access to motorized transportation.

## Introduction

The relationship between neighborhood food environments and obesity occupies a central role in policy debates (1–4). A recurring theme is the notion of “food deserts,” where access to healthful and affordable food is limited (5,6). Food deserts are often identified by the absence of supermarkets or full-range grocery stores and by the presence of fast-food restaurants and convenience stores (6). Hypotheses that link the food environment with obesity claim that proximity to fast-food outlets, convenience stores, or small grocery stores undermines diet quality, whereas proximity to supermarkets or full-range grocery stores enhances it by providing healthful products, mainly fruits and vegetables (7). However, evidence is more tentative than often presented in the news media and in policy arguments (7,8). Associations between food environment measures and obesity are not reliably replicated, and dietary behaviors are not consistent with these associations (9). Varying definitions of “neighborhood” could also contribute to the inconsistency of results and lack of comparability of data. Few studies have examined the association between varying neighborhood sizes and dietary intake and weight status (10–12). The objective of this study was to examine the associations of number and type of neighborhood food environments with dietary intake and body mass index (BMI) measures by using definitions of “neighborhood” based on various geographic sizes. We analyzed data from the California Health Interview Survey.

## Methods

We used data from the 2007 and 2009 waves of the California Health Interview Survey (CHIS), a random-digit–dial telephone survey of California’s noninstitutionalized population (13). In 2007 and 2009, the CHIS included interviews of 98,662 adults aged 18 years or older. The sampling weights provided by CHIS account for unequal sampling probabilities and nonresponse to allow generalization to the study population. We limited our analysis to 97,678 adults aged 18 years or older, excluding 533 (0.5%) pregnant women and 451 (0.5%) respondents whose information was provided through a proxy interview.

Self-reported measures on dietary intake included the number of times per week the following were consumed during the month before the interview: fruits (excluding juices), vegetables (excluding fried potatoes), sugar-sweetened soft drinks (excluding diet soft drinks), and fried potatoes (including French fries, home fries, and hash browns). The measures also included the number of times fast food had been consumed in the week before the interview. The information was collected through questions in the following format: “During the past month [‘or in the past 7 days’ for fast-food consumption], how often did you eat [food item name]?” The question was often followed by clarification of the food items. The response was standardized to reflect mean per-week intake frequency.

Anthropometric measures included BMI, calculated as self-reported weight in kilograms divided by self-reported height in meters squared. We defined “overweight or obese” as a BMI of 25.0 or more and “obese” as a BMI of 30.0 or more, according to World Health Organization classifications for adults (14).

Whereas most large survey studies use a predefined administrative unit such as census tract or zip code to define neighborhood, we defined neighborhood on the basis of geographic distance from a respondent’s residence, and we defined neighborhood food environment by counting the number of different types of food outlets within those distances. We drew circular buffers of varying radii (0.25, 0.5, 1.0, 1.5, and 3.0 miles) centered on each respondent’s residential address. Because 1 mile is often used as a threshold for walkable distance (6), we considered distances of 0.25, 0.5, and 1.0 miles to be within walking distance. We measured Euclidean distance (straight line distance between 2 points) using ArcMap 9.1 (ESRI, Redlands, California).

We used food outlet data from the 2008 release of InfoUSA (15). We overlaid food outlet locations on the buffers around respondents’ residences and counted the number of different types of food outlets in each buffer. We classified fast-food restaurants, full-service restaurants, convenience stores, small food stores, mid-size grocery stores, and large supermarkets by using the North American Industry Classification System (NAICS) (16). NAICS does not have a code for fast-food restaurants; we identified 63 major fast-food franchises that have main menus that include items such as hot dogs, hamburgers, pizza, fried chicken, submarine sandwiches, or tacos by NAICS codes 72221105–6. Full-service restaurants were identified by NAICS codes 72211001–20; convenience stores, code 44512001; and small food stores (annual sales <$1 million), mid-size grocery stores (annual sales of $1–$5 million), and large supermarkets (annual sales >$5 million), codes 44511001–3, respectively.

To examine the association between neighborhood food environment and dietary intake, we performed negative binomial regression analysis. The dietary intake measures were the dependent variables, and the numbers of different types of food outlets in the buffers were the explanatory variables. Negative binomial regression is a generalization of the Poisson model in which the Poisson parameter has a random component (17). We performed separate regressions for each dietary intake measure and buffer size, calculated average marginal effects (AMEs), which measure an estimated change in the outcome in the observed unit associated with 1 unit change in the regressor of interest, and applied the Bonferroni adjustment for multiple comparisons. We controlled for potentially confounding individual and neighborhood factors. Individual-level control variables included sex, age (in years and age squared), race/ethnicity (white, African American, Hispanic, Asian or Pacific Islander, Native American, other race/multirace), household size, annual household income (in natural logarithm), education (not a high school graduate, high school graduate, high school graduate but not college graduate, college graduate, and more than college degree), marital status (married, divorced/separated/widowed, single), parental status (has a child or has no child), physical activity (regular activity, some activity, sedentary), and survey year. Although we were interested in vehicle ownership, this information was not collected in CHIS 2009 and therefore not included in our analysis. Proxies for neighborhood-level control variables, which were obtained from 2000 Census data (18), included population density, median household income, and proportion of non-Hispanic white residents of a respondent’s residential census tract. These factors did not match our definition of neighborhood for the explanatory variables, but they could be derived only from predefined administrative units.

To examine the association between the neighborhood food environment and BMI measures, we regressed BMI (through ordinary least squares [OLS]) and its dichotomous cutoffs of BMI of 25.0 or more and BMI of 30.0 or more (through logistic regression) on the same set of explanatory and control variables. Again, we performed separate regressions for each buffer size, calculated AMEs for logistic regression models, and applied the Bonferroni adjustment for multiple comparisons. We then compared the significant associations of food environments with dietary intakes and BMI measures to examine the hypothesis that the neighborhood food environment influences BMI through its influence on dietary intakes.

We performed several sensitivity analyses: stratified analysis by urbanicity (urban vs nonurban residents) and income level (federal poverty level [FPL] ≤130% vs FPL >130%) and analysis by density (instead of raw counts) of food outlets by census tract (the number of food outlets in census tract per 1,000 population and the number of food outlets in census tract per square mile). We defined “low income” as FPL of 130% or less. All analyses were performed in Stata 12.1 (StataCorp LP, College Station, Texas). We weighted the regression using sampling weights and estimated *P* values based on heteroscedasticity-robust standard errors obtained by using the Eicker–Huber–White sandwich estimator.

## Results

Of the dietary items surveyed, mean intake was greater for fruits (7.7 times per week) and vegetables (6.9 times per week) than for sugar-sweetened soft drinks (2.2 times per week), fast food (1.5 times per week), or fried potatoes (0.9 times per week) (Table 1). Average BMI was 26.8; 57% of respondents were overweight or obese, and 22.7% were obese.

**Table 1:** Characteristics of Respondents and Census Tracts, California Health Interview Survey (CHIS), 2007 and 2009^a^

Characteristic	Value
**Survey Respondents**	
**Dietary intake, mean (IQR), no. of times consumed per week**	
Sugar-sweetened soft drinks	2.2 (0–2)
Fast food	1.5 (0–2)
Fruits	7.7 (3–14)
Fried potatoes	0.9 (0–1)
Vegetables	6.9 (3–7)
**Body weight**	
BMI, mean (SD), kg/m^2^	26.8 (5.8)
Obese (BMI ≥30.0), no. (%)	21,682 (22.7)
**Male, no. (%)**	39,690 (49.5)
**Age**	
Age, mean (SD), y	45.0 (17.4)
Age squared, mean (IQR)	2,328 (961–3249)
**Race/ethnicity, no. (%)**	
White, non-Hispanic	65,437 (48.8)
African American, non-Hispanic	4,416 (5.9)
Asian or Pacific Islander, non-Hispanic	9,331 (13.3)
Native American, non-Hispanic	1,135 (0.9)
Other race or multirace, non-Hispanic	5,868 (8.6)
Hispanic	11,491 (22.5)
**No. of household members, mean (IQR)**	3.3 (2–4)
**Natural logarithm of annual household income, mean (SD)**	10.7 (1.5)
**Education, no. (%)**	
Not a high school graduate	9,623 (16.2)
High school graduate	21,562 (26.5)
High school graduate but not college graduate	19,900 (17.6)
College graduate	29,363 (26.5)
More than college degree	17,230 (13.2)
**Marital status, no (%)**	
Married	55,405 (61.7)
Divorced/separated/widowed	27,571 (14.4)
Single	14,702 (24.0)
**Have a child, no. (%)**	25,206 (33.9)
**Physical activity level, no. (%)**	
Sedentary	24,042 (24.2)
Some activity	45,240 (46.7)
Regular activity	28,396 (29.1)
**CHIS participants interviewed in 2009, no. (%)**	47,114 (50.6)
**Urban residence, no. (%)**	79,180 (88.9)
**Census Tracts^b^**	
Population per square mile, mean (IQR), no.	8,025 (2,398–10,151)
Household income, median (SD), $	69,127 (34,346)
Proportion of non-Hispanic whites, mean (SD), %	56.5 (21.1)

Abbreviations: IQR, interquartile range; BMI, body mass index; SD, standard deviation.

a Percentages and means are weighted using CHIS sampling weights. Counts are unweighted. Percentages may not sum to 100 because of rounding.

b Data source: US Census Bureau (18).

The number of food outlets per food outlet type in each buffer varied in the expected way: we found more small food stores and fast-food restaurants on average than other food outlet types (Table 2); large supermarkets and mid-size grocery stores were less common. We found an average of approximately 7 fast-food restaurants, 3 full-service restaurants, 2 convenience stores, 7 small food stores, 1 mid-size grocery store, and 2 large supermarkets in the 1-mile buffer. Low-income respondents were surrounded by more food outlets of each type, including mid-size grocery stores and large supermarkets, than were their wealthier counterparts.

**Table 2:** Number of Food Outlets by Type, Buffer Size^a^, and Federal Poverty Level (FPL), California, 2007 and 2009

Food Outlet Type, by Buffer	No. of Outlets, Mean (95% CI)	Mean No. of Outlets^b^	
		FPL ≤130%	FPL >130%
**0.25-Mile radius**			
Fast-food restaurant	0.45 (0.43–0.47)	0.57	0.42
Full-service restaurant	0.18 (0.17–0.19)	0.24	0.17
Convenience store	0.15 (0.15–0.16)	0.22	0.14
Small food store	0.56 (0.53–0.59)	1.02	0.44
Mid-size grocery store	0.09 (0.08–0.10)	0.16	0.07
Large supermarket	0.13 (0.12–0.13)	0.15	0.12
**0.5-Mile radius**			
Fast-food restaurant	1.92 (1.88–1.97)	2.31	1.82
Full-service restaurant	0.74 (0.71–0.76)	0.84	0.71
Convenience store	0.57 (0.56–0.58)	0.80	0.51
Small food store	2.05 (1.96–2.15)	3.45	1.69
Mid-size grocery store	0.33 (0.32–0.35)	0.52	0.28
Large supermarket	0.50 (0.49–0.51)	0.56	0.48
**1.0-Mile radius**			
Fast-food restaurant	7.34 (7.24–7.45)	8.53	7.03
Full-service restaurant	2.65 (2.60–2.70)	2.96	2.57
Convenience store	1.99 (1.95–2.03)	2.62	1.82
Small food store	7.07 (6.80–7.34)	11.32	5.95
Mid-size grocery store	1.14 (1.11–1.18)	1.73	0.99
Large supermarket	1.82 (1.80–1.85)	2.01	1.78
**1.5-Mile radius**			
Fast-food restaurant	15.24 (15.06–15.42)	17.45	14.66
Full-service restaurant	5.36 (5.28–5.44)	5.98	5.20
Convenience store	4.02 (3.95–4.08)	5.15	3.72
Small food store	14.29 (13.82–14.76)	22.37	12.17
Mid-size grocery store	2.31 (2.25–2.37)	3.33	2.05
Large supermarket	3.70 (3.66–3.75)	4.03	3.62
**3.0-Mile radius**			
Fast-food restaurant	49.91 (49.38–50.45)	57.38	47.96
Full-service restaurant	16.92 (16.73–17.12)	18.95	16.39
Convenience store	12.89 (12.72–13.06)	16.11	12.05
Small food store	45.91 (44.61–47.21)	69.56	39.72
Mid-size grocery store	7.32 (7.17–7.47)	9.99	6.62
Large supermarket	11.84 (11.70–11.97)	12.90	11.56

Abbreviation: CI, confidence interval.

a Means are weighted using California Health Interview Survey sampling weights. Standard errors were obtained through Taylor series linearization and account for sampling weights and stratification. Buffer size was determined by measuring the distance from the survey respondent’s residence.

b All differences are significant (*P* < .001), based on 2-sample *t* test with unequal variances. The federal poverty level, issued annually by the US Department of Health and Human Services, is determined by household income and the number of household members.

Among all 5 buffer sizes, most (79% [119/150]) of the effects of the number of food outlets on intake of food items were not significant. Eight effects were significant after applying Bonferroni’s adjustment; 7 of these were clustered around larger buffer sizes (1.5 miles and 3.0 miles) and fast-food restaurants and large supermarkets (Table 3). The number of fast-food restaurants in the 3.0-mile buffer was positively associated with an increase in intake frequency of sugar-sweetened soft drinks, fast food, and fried potatoes, and less frequent consumption of fruits and vegetables. By contrast, the number of large supermarkets within 1.5- and 3.0-mile buffers was associated with less frequent consumption of sugar-sweetened soft drinks.

**Table 3:** Estimated Change (Average Marginal Effect [AME])^a^ in Intake^b^ of Food Item, By Food Outlet Type and Food Environment^c^, California, 2007 and 2009

Food Item, by Food Outlet Type	Buffer Size (Radius)					
	1.0 Mile		1.5 Mile		3.0 Mile	
	AME	*P* Value^d^	AME	*P* Value^d^	AME	*P* Value^d^
**Fast-food restaurant**						
Fruits	−0.023	.14	−0.022	.02	−0.014	.001
Vegetables	−0.019	.20	−0.025	.01	−0.020^e^	<.001
Sugar-sweetened soft drinks	0.057	.02	0.022	.001	0.015^e^	<.001
Fast food	0.015^e^	<.001	0.020^e^	<.001	0.013^e^	<.001
Fried potatoes	0.006	.16	0.005	.03	0.005^e^	<.001
**Full-service restaurant**						
Fruits	0.034	.33	0.046	.05	0.011	.36
Vegetables	0.040	.30	0.046	.06	0.029	.003
Sugar-sweetened soft drinks	−0.040	.18	0.010	.63	0.004	.71
Fast food	−0.026	.03	−0.030	.001	−0.012	.003
Fried potatoes	−0.010	.20	−0.010	.09	−0.006	.06
**Convenience store**						
Fruits	0.029	.43	−0.013	.60	0.004	.76
Vegetables	0.001	.98	0.012	.57	0.009	.41
Sugar-sweetened soft drinks	−0.013	.67	0.011	.52	0	.99
Fast food	0.005	.72	0.005	.61	−0.008	.03
Fried potatoes	0.002	.79	0.007	.27	−0.001	.85
**Small food store**						
Fruits	−0.004	.56	−0.004	.41	−0.003	.13
Vegetables	−0.001	.93	−0.004	.36	−0.002	.27
Sugar-sweetened soft drinks	0.003	.61	0.001	.84	0	.90
Fast food	0.003	.47	0.001	.74	0.001	.12
Fried potatoes	0	.83	0	.73	0.001	.22
**Mid-size grocery store**						
Fruits	0.032	.48	0.026	.42	0.025	.12
Vegetables	0.031	.54	0.012	.71	0.008	.56
Sugar-sweetened soft drinks	−0.016	.62	−0.038	.20	−0.022	.19
Fast food	−0.054	.001	−0.021	.14	−0.016	.02
Fried potatoes	0.007	.53	−0.001	.90	−0.004	.36
**Large supermarket**						
Fruits	−0.010	.83	0.025	.44	0.028	.05
Vegetables	−0.013	.75	0.005	.85	0.018	.14
Sugar-sweetened soft drinks	−0.130	<.001	−0.106^e^	<.001	−0.051^e^	<.001
Fast food	−0.026	.10	−0.030	.003	−0.020	.002
Fried potatoes	−0.013	.25	−0.013	.07	−0.010	.009

Abbreviations: SD, standard deviation; NA, not applicable.

a AMEs measure an estimated change in the per-week frequency of consumption of each food item associated with 1 unit change in the regressor of interest.

b Number of times the item was consumed per week.

c Food environment was defined by counting the number of food outlet types (eg, supermarket) in each buffer of a certain radius (eg, 1.0 mile) centered on a respondent’s residence. Statistics were adjusted by using California Health Interview Survey sampling weights.

d *P* values were calculated by using *z* statistic obtained through negative binomial regression and based on standard errors estimated using the Eicker–Huber–White sandwich estimator.

e AME is different from zero (at .05 level) after applying Bonferroni’s adjustment for multiple comparisons. All 6 food outlet types were included in the regression models, and individual- and census tract–level characteristics were controlled for (but are not presented here).

Among all 5 buffer sizes, most (83% [75/90]) of the effects of the number of food outlet types on BMI, and weight status of a BMI of 25.0 or more or a BMI or 30.0 or more were not significant. Seven effects were significant after applying Bonferroni’s adjustment; these were significant in the larger buffer sizes and large supermarkets and fast-food restaurants. The number of fast-food restaurants in the 3.0-mile buffer was positively associated with the probability of a BMI of 25.0 or more but not with BMI or the probability of a BMI of 30.0 or more (Table 4). The number of large supermarkets in the 3.0-mile buffer was associated with lower BMI and lower probabilities of a BMI of 25.0 or more and a BMI of 30.0 or more. Some of these associations were also found in the 1.5- and 1.0-mile buffers.

**Table 4:** Estimated Change (Average Marginal Effect [AME]^a^) in Body Mass Index (BMI) and the Probability of Overweight or Obesity, by Food Outlet Type and Food Environment, California, 2007 and 2009^b^

Food Outlet Type/BMI Measure	Buffer Size (Radius)					
	1.0 Mile		1.5 Mile		3.0 Mile	
	AME	*P* Value^c^	AME	*P* Value^c^	AME	*P* Value^c^
**Fast-food restaurant**						
BMI	0.020	.07	0.010	.17	0.009	.02
BMI ≥25.0	0.001	.23	0.001	.11	0.001^d^	<.001
BMI ≥30.0	0.001	.35	0	.62	0.001	.006
**Full-service restaurant**						
BMI	−0.059	.02	−0.014	.47	0.010	.27
BMI ≥25.0	−0.003	.26	−0.001	.48	0	.59
BMI ≥30.0	−0.003	.12	0	.92	0	.91
**Convenience store**						
BMI	0.007	.79	−0.005	.78	0.003	.73
BMI ≥25.0	−0.002	.34	−0.002	.18	−0.001	.31
BMI ≥30.0	0.002	.19	0.001	.47	0	.65
**Small food store**						
BMI	0.005	.47	0.001	.86	−0.001	.32
BMI ≥25.0	0	.91	0	.54	0	.82
BMI ≥30.0	0	.39	0	.89	0	.83
**Mid-size grocery store**						
BMI	−0.029	.44	−0.002	.93	0	>.99
BMI ≥25.0	−0.003	.34	−0.003	.19	0	.79
BMI ≥30.0	−0.005	.04	−0.002	.30	−0.001	.42
**Large supermarket**						
BMI	−0.115^d^	<.001	−0.093^d^	<.001	−0.073^d^	<.001
BMI ≥25.0	-0.008	.003	-0.008^d^	<.001	-0.005^d^	<.001
BMI ≥30.0	−0.007	.001	−0.006	.001	−0.005^d^	<.001

a AME on BMI reflects the estimated change in BMI (in kg/m^2^); AME on BMI ≥25 (or BMI ≥30) reflects the estimated change in the probabilities of being overweight or obese (or of being obese) associated with 1 unit change in the regressor of interest.

b Food environment was defined by counting the number of food outlet types (eg, supermarket) in each buffer of a certain radius (eg, 1.0 mile) centered on a respondent’s residence. Statistics were adjusted by using California Health Interview Survey sampling weights.

c *P* value for BMI was calculated by using *t* statistic obtained through ordinary least squares regression. *P* value for BMI ≥25 and BMI ≥30 was calculated by using *z* statistic obtained through logistic regression and based on standard errors estimated using the Eicker–Huber–White sandwich estimator.

d AME is different from zero (at .05 level) after adjustment for multiple comparisons. All 6 food outlet types were included in the regression models, and individual- and census tract–level characteristics were controlled for (but are not presented here).

More fast-food restaurants in the 3.0-mile buffer predicted increased frequency of consuming fried potatoes, sugar-sweetened soft drinks, and fast food, decreased frequency of consuming vegetables, and a lower probability of a BMI of 25.0 or more. By contrast, the number of supermarkets was largely not associated with dietary intake, whereas more supermarkets within 1.0-, 1.5-, and 3.0-mile buffers predicted lower BMI.

When we stratified the analysis by urbanicity and FPL, we did not find the significant associations between the number of food outlet types and dietary intake or BMI measures among nonurban respondents and low-income respondents that we observed in unstratified analysis, but we did find them among their urban and wealthier counterparts.

When we used census tract data, only the association between the number of mid-size grocery stores in census tract per 1,000 population and fast-food consumption was significant after adjustment for multiple comparisons; we found no significant associations for the other food outlet types or BMI measures.

## Discussion

Our study did not find strong evidence to support the hypothesis that the food environment within walkable distance is associated with the dietary intake or BMI measures of residents. We found some evidence that the food environment for larger, nonwalkable areas (1.5- and 3.0-mile buffers) is associated with dietary intake and BMI measures. Moreover, significant results for BMI measures do not match dietary intakes, except for the number of fast-food restaurants within a 3.0-mile buffer, which was simultaneously associated with increased frequency of consuming fried potatoes, sugar-sweetened soft drinks, and fast food, decreased frequency of consuming vegetables, and greater probability of being overweight or obese (a BMI of 25.0 or more). We found an association between BMI measures and supermarkets only for the 1.5- and 3.0-mile buffers, but those associations did not parallel dietary intake. In particular, we found no association between supermarkets and vegetable and fruit consumption at any buffer size. When we compared the density of food outlets on the basis of residential census tract instead of raw counts, we observed only 1 significant association (between the number of mid-size grocery stores in census tract per 1,000 population and fast-food consumption) and there were no significant associations between other types of food outlets and either BMI measures or dietary intake. Therefore, our data do not confirm previous data, especially data on the association between fast-food restaurants or supermarkets and dietary intake or BMI. The primary strength of this study is that we explored a range of distances from respondents’ residential address, from areas smaller than census tracts to areas much larger, instead of using predefined units, such as census tracts or zip codes.

This study has several limitations. First, we measured food outlet type, not the availability of certain foods. We obtained food outlet data from InfoUSA; business listings are rarely up-to-date or without error. One study reported only fair agreement between commercial data and field observations for supermarkets, grocery stores, convenience stores, and full-service restaurants and poor agreement for fast-food restaurants (19). The dietary intake questions we used did not provide guidance on serving size, and they asked about a small number of food items; hence, they did not allow examination of overall diet quality. The questions also had a long recall period of 1 month. BMI was calculated from self-reported height and weight; self-report tends to underestimate BMI (20–22). The study used data from California, and the results may not apply to other geographic regions or populations. Finally, this study used cross-sectional data and was unable to establish causation.

The limitations of the data used in this study may have contributed to our null findings, but there are other, substantive reasons for finding a null relationship between of the number of food outlet types and dietary intake or BMI. A likely reason for our null findings is that food-shopping patterns are weakly related, if at all, to neighborhoods in the United States because of access to motorized transportation. Access to motorized transportation increases mobility and may limit the relevance of immediate food environments by allowing residents to travel beyond a walking distance. California has one of the highest rates of vehicle ownership (23); most of its population resides in metropolitan areas (24). Although the local food environment may be important for rural households (where residents are sparsely distributed) or households that lack access to motorized transportation, the sample size in our study may have been too small to detect associations affected by rural residence or car ownership. The most recent CARDIA study reports associations between fast-food restaurants in immediate neighborhoods and fast-food consumption among low-income men but not for higher-income groups or neighborhoods outside walking distance, suggesting greater reliance on immediate food environments among low-income people who are less likely to own a car (11).

Food environment research has often been based on predefined administrative units, typically census tracts, which correspond to our smaller buffer sizes. In California, the median census tract corresponds to a buffer with a 0.5-mile radius; nationwide, the median census tract corresponds to a buffer with a 0.79-mile radius, both within the walkable distance of 1 mile (6). However, the 1-mile definition may no longer be relevant in the motorized society of the United States and California. Further research on varying sizes of neighborhoods is warranted to understand people’s food-shopping travel patterns and how the patterns expose people to different food environments.

In the literature of food deserts, it is often argued that lower-income communities have reduced access to foods. However, these results are often based on density measures (eg, stores per capita in census tract) (25,26). When we compared the density of food outlets among low- and higher-income respondents by using data on density based on census tracts (the number of food outlets in census tract per 1,000 population), the densities of supermarkets and full-service restaurants were lower among low-income people. However, low-income areas are also much more densely populated, so the distance between a residence and a food outlet may be a more relevant criterion than density. In our definitions of neighborhoods, which used circular buffers centered on individuals’ homes, neighborhood sizes are identical for all individuals and the dependent variable (the number of food outlet types per buffer) has the same physical meaning. Using this uniformly defined measure, we found that low-income respondents had greater access to each type of food outlet. Our findings are consistent with a report by the US Department of Agriculture, which found a smaller median distance to the nearest supermarket among low-income individuals (compared with higher-income individuals) in urban areas (6). Although predefined administrative units (eg, census tracts) may be more feasible for monitoring purposes, measures reflecting the proximity of food outlets may more reliably identify disadvantaged communities.

The concept of neighborhood food environments has been the focus of the news media and policy makers, yet the evidence is not clear on whether promoting or discouraging a particular type of food outlet is an effective approach to promoting healthful dietary behaviors and weight status. Initial findings in a new area of research — such as food environments — may be qualified over time, and both exact replication and conceptual replication of previous findings using alternative data sources and methods is a central theme for advancing scientific knowledge and informing policies. No single study completely addresses a research question, and this study can only contribute another data point. However, at least in California, the relationship between neighborhood food outlets and dietary intake or BMI is subtler than the relationship presented in the news media. The relationship appears to exist in larger geographic areas rather than within walking distances or in a typical urban census tract, larger areas that are arguably outside what we consider to be a neighborhood.
